# Supplementary material for: BMP-2/β-TCP Local Delivery for Bone Regeneration in MRONJ-Like Mouse Model
Source: Int J Mol Sci. 2020 Sep 24;21(19):7028. doi: 10.3390/ijms21197028 (PMC7583034; doi:10.3390/ijms21197028)
Supplement: Supplementary file 1 [file ijms-21-07028-s001.pdf]

Supplementary Material

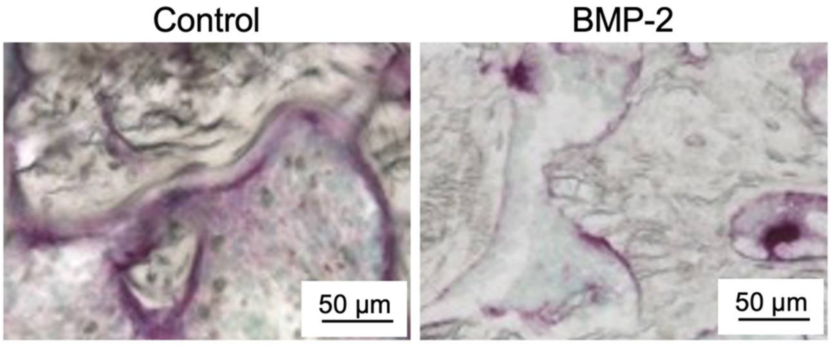

Figure S1. Tartrate-resistant acid phosphatase (TRAP) staining of the regenerated bone in the tooth extraction socket. TRAP staining was performed according to standard protocol, with histological sections from specimens collected four weeks after transplantation of BMP-2/β-TCP in the MRONJ treatment model. TRAP-positive osteoclasts were observed on the regenerated bone surface in both the BMP-2/β-TCP transplantation and non-transplantation control groups.
